# Supplementary material for: Subyearling Chinook salmon diets in Lower Columbia River estuarine habitats
Source: PLoS One. 2025 Jun 12;20(6):e0325939. doi: 10.1371/journal.pone.0325939 (PMC12161559; doi:10.1371/journal.pone.0325939)
Supplement: Attachment — Submitted filename: Response to Reviewers.docx [file pone.0325939.s002.docx]

Thank you for taking the time to thoroughly review our manuscript. We greatly appreciate your input and have responded below.

**Response to Academic Editor:**

1. Please ensure that your manuscript meets PLOS ONE's style requirements, including those for file naming. Response: we removed spaces from figure file names, corrected Level 2-3 headings, bolded only figure titles, fixed Table captions (moving legend below table and bolding only title), fixed references including replacing ‘et al.’ with all authors past author 6.

2. To comply with PLOS ONE submissions requirements, in your Methods section, please provide additional information regarding the experiments involving animals and ensure you have included details on (1) methods of sacrifice, and (2) efforts to alleviate suffering. We have added “Sampled fish were maintained in buckets with oxygenated, in situ water, with ice added, as necessary, to maintain water temperatures. Trained biologists quickly euthanized fish with a lethal dose of MS-222, placed them on ice to cold shock to slow digestion and for additional analyses not described here.”

3. We note that the grant information you provided in the ‘Funding Information’ and ‘Financial Disclosure’ sections do not match. When you resubmit, please ensure that you provide the correct grant numbers for the awards you received for your study in the ‘Funding Information’ section. We have changed our information to “This study was funded under Project# 2003-007-00 between the Lower Columbia Estuary Partnership (LCEP) and the Northwest Power and Conservation Council/Bonneville Power Administration, to support the Ecosystem Monitoring Program (implemented by LCEP), and inform regional habitat restoration efforts.”.

4. see Cover Letter.

5. Please include your full ethics statement in the ‘Methods’ section of your manuscript file. In your statement, please include the full name of the IRB or ethics committee who approved or waived your study, as well as whether or not you obtained informed written or verbal consent. If consent was waived for your study, please include this information in your statement as well. Our study protocols were reviewed and granted written approval through our federal permit (NOAA Section 10 #22944-2R), which serves as the ethics board following Institutional Animal Care and Use Committee guidelines.

6. We note that you have indicated that there are restrictions to data sharing for this study. PLOS only allows data to be available upon request if there are legal or ethical restrictions on sharing data publicly…We uploaded our data on Dryad on 4.09.25. The DOI is now active. In the Data Availability section, we have included “Data used in this study is available at <https://doi.org/10.5061/dryad.c59zw3rm2>. To date LCEP EMP data is visualized at <https://public.tableau.com/app/profile/ecosystem.monitoring.program/viz/MacroinvertebrateCommunities/WelcometotheMacroinvertebrateEcosystemMonitoringDashboard>”

7. We note that Figure 1 in your submission contain [map/satellite] images which may be copyrighted. All PLOS content is published under the Creative Commons Attribution License (CC BY 4.0), which means that the manuscript, images, and Supporting Information files will be freely available online, and any third party is permitted to access, download, copy, distribute, and use these materials in any way, even commercially, with proper attribution…We modified our Fig 1 to be a stylized Mapbox basemap instead of a satellite basemap, and obtained written permission (file uploaded) from Mapbox, who approved the use of the image under CC by 4.0. We were not required to have permission from OpenStreetMap.

8. Please review your reference list to ensure that it is complete and correct. If you have cited papers that have been retracted, please include the rationale for doing so in the manuscript text, or remove these references and replace them with relevant current references. Any changes to the reference list should be mentioned in the rebuttal letter that accompanies your revised manuscript. If you need to cite a retracted article, indicate the article’s retracted status in the References list and also include a citation and full reference for the retraction notice. We reviewed our reference list and aside from minor modifications to author name positioning and reducing author names after 6^th^ author to ‘et al.’, we believe our list is correct and there are no papers cited that have been retracted.

**Response to Reviewer #1: Review of PONE-d-25-06245**
Key words: consider the overlap between key words and title. The authors may want to change some of the key words to have a wider search response. Response: we have modified our key words to include more specific diet vocabulary including prey energy, amphipods, cladocerans, and dipterans.

L13: “is” should be past tense “was” to match the rest of the sentence. Corrected.

L17: “river discharge” should have some more information. Maintaining adequate river discharge? What sort of river discharge. Sample with L18 connectivity. Increased connectivity? Changed to “Juvenile salmon rearing resiliency in the estuary can be aided by maintaining sufficient river discharge levels for salmon, and by restoring habitat and habitat connectivity to the mainstem channel.”

Fig 1 should have a scale bar. It might also be useful for international readers if there were an inset of a broader geographical region. That part is optional but might help people interpret the location of the LCRE. Figure 1 has a scale bar in the lower righthand corner.

L103 and L107 spell Campbell differently Corrected.

L117: this would be a good point to mention the IACUC protocols used and collection permits We included “Juvenile Chinook salmon were collected under Washington State Collection Permits RCW 77.15.660 and WAC 220-200-04, and under NMFS Northwest Fisheries Science Center Scientific Research Permit 22944. Sampled fish were maintained in buckets with oxygenated, in situ water, with ice added, as necessary, to maintain water temperatures. Trained biologists quickly euthanized fish with a lethal dose of MS-222, placed them on ice to potentially cold shock (IUCAC approved), to slow digestion, and for additional analyses not described here.

L118: what level were prey identified to? Genus? Broader taxonomic groupings? I see on L119 it says family, genus, or species. How was that decided? Lowest possible taxonomic unit? Did it vary depending on whether it was a macroinvertebrate or zooplankter? Was the entire stomach contents counted or a fraction of it and then mathematically scaled to the whole thing? Changed to “In the laboratory, entire stomachs were analyzed, and each prey taxon was counted, blotted dry, weighed to the nearest 0.0001 g, and identified to the finest possible taxonomic unit (crustaceans to genus or species and insects to family or order).” Note to reviewer: if the prey is heavily digested, we identify to the broader taxonomic level, but entire stomach contents are analyzed.

Methods: there is no information about how the water quality data were collected. The first part of the results includes temperatures, but the reader is not sure whether these are surface temperatures, buoy data, or continuous loggers deployed at some reasonable sampling depth. L127 Changed to “Water quality has been continuously measured at EMP sites using Yellow Springs Instruments (YSI) equipped with water temperature, specific conductance, pH, and dissolved oxygen probes.”

Results: the water temperature is interesting, but sort of an island. Maybe a figure if they are continuous monitoring data would be prudent? I see it in Figure 6 now that I’ve reached that part, but it is not referenced in text L177-181 where it’s mentioned. And Fig 6 also doesn’t include Fraz Lake and Ilwaco Slough. We changed Fig 6 to Fig 2 so it is referenced with temperature results, which also indicate that 3 of the sites had highest salmon densities. We also moved our maximum optimal water temp from 15.5 to 17.5, to reflect WA Ecology parameters. We feel this parameter (17.5) is more consistent with other literature, including research by Dan Bottom and Oregon Dept. of Ecology standards.

All figures: I printed the manuscript to read and the colors are not distinguishing very well in black and white. Are the colors selected for the figures color-blind friendly? R has many great options for printer and color-blind friendly palettes. The authors could check out colorbrewer2.org or the viridis color schemes in the ‘viridis’ R package. Fig 5 uses a colorbrewer theme, however the ‘stomach fullness’ ranges for each color are not defined in the legend, which would be helpful for the reader. Changed Fig 5 to add stomach fullness ranges in legend. We did not change color palettes for other figures because our colors (i.e. for sites and prey taxa) match the colors chosen for LCEP EMP Tableau visualizations and we want color consistency across datasets.

For Fig 2, it is hard to interpret the lines. The authors could consider stacked bar graphs here to show the relative importance of different prey items. I can see that Amphipoda and Diptera seem to be the most important and fish and Trichoptera the least, but stacked bar graphs may be more interpretable. We have changed Fig 2 from line plots to stacked bar plots for easier interpretation.

For Fig 5, it is a bit unclear whether the y-axis “Juvenile Chinook salmon counts” means the number of stomachs assessed or some sort of population per unit area estimate. The latter would be really interesting to see if stomach fullness was related to competition. We modified the plot y-axis label to “Juvenile Chinook Salmon” to indicate the salmon are our diet stomachs.

L433-439 seem tangential and should not be the final information a reader gets from this manuscript. The authors should move this to a different part of the discussion and end on their most major and broad-sweeping conclusions, or eliminate it all together. We agree and have eliminated the final lines of the manuscript.

**Response to Reviewer #2: Review of PONE-d-25-06245**
General comments
I thought this paper was a well-written summary of a long-term study investigating juvenile Chinook salmon diets in the heavily managed Columbia River estuary. The statistical analysis, tables, and figures were appropriate.
I have two major concerns with the paper. First, the authors don’t present testable hypotheses embedded in ecological theory to drive the analysis. As it stands, the paper is just a statistical description of the data. Second, there was no attempt to correlate river discharge or climate indices (ENSO) in driving some of temporal variation in diet composition despite having a 14 year data set. Response: the purpose of our manuscript is a long-term statistical description of juvenile Chinook diets in the Lower Columbia estuary, including identifying potential diet trends changes associated with differing water temperatures. Diets currently appear stable. However, we intend to conduct bioenergetics analyses that test how potential increased water temperature regimes change juvenile salmon prey assemblages and salmon metabolic costs. Other manuscripts are forthcoming among EMP researchers that focus on water levels and sediment in the Lower Columbia (dam, freshet), vegetation, and prey and fish assemblages.

Specific comments
Line 30: I suggest adding more details about the Columbia River here after you first mention the system. Because we don’t introduce the Columbia River until the following paragraph, we removed the reference to it.

Line 52: I suggest adding detail about the LCEP here. We moved the introduction of the LCEP two paragraphs down, where we discuss the partnership in detail. The intro sentence of the paragraph is now “Shortly after the LCRE was deemed an estuary of national significance, the Lower Columbia Estuary Partnership (LCEP) was established.”

Line 97: Please define ‘good water quality’. We removed the 2 references to ‘good water quality’ and kept the references to the LCEP EMP annual report, which highlights ‘good’ water quality but does not define it.

Page 118-119: How did you deal with unidentifiable items? I’ve analyzed a lot of fish diet samples and there is a lot of body parts, etc that are not easily identifiable without DNA analysis. This material can dominate the stomachs of some fish. What about fish with empty stomachs? 1. Juvenile salmon diets are rarely, if ever, dominated by unidentifiable material, mainly because we analyze stomach (foregut) contents and do not count intestine (hindgut) contents.  In cases where there is unidentifiable material, we label it as "animal matter". 2. L193 added ‘18 fish with empty stomachs were removed from analyses.’

Line 176: Why no data on discharge? Seems like this is an important driver of diets and fish populations but it was not explicitly evaluated despite being mentioned a lot by the authors. A manuscript by other EMP research scientists related to river flow, discharge, freshet levels, and temperature is in prep.

Line 402: You did not assess fish growth so you cannot say your study provides insight into how tidal wetlands influence this process. We removed the statement that our results verified the importance of extant tidal wetlands for growth.

Finally, we have made a few additional changes to the manuscript. 1. We have added Jeffrey Grote as a co-author. He is the EMP scientist that collects fish and neuston samples for our diets study. He has reviewed our revised manuscript. 2. We changed the black horizontal line in the new Fig 2 that represents optimum water temps for juvenile salmon from 15.5^°^ to 17.5^°^. The change better reflects LCRE literature on juvenile Chinook.

Thank you.
